# Depression and Suicidality in Multiple Sclerosis: Red Flags, Management Strategies, and Ethical Considerations

**DOI:** 10.1007/s11910-019-0992-1

**Published:** 2019-08-28

**Authors:** Rosalind Kalb, Anthony Feinstein, Amanda Rohrig, Lauren Sankary, Alissa Willis

**Affiliations:** 1 West Bath, ME USA; 2 Can Do Multiple Sclerosis, Edwards, Avon, CO USA; 3 Department of Psychiatry, University of Toronto and Sunnybrook Health Sciences Centre, Toronto, Canada; 4 Horizon Rehabilitation Centers, Omaha, NE USA; 5 Cleveland Clinic Center for Bioethics, Cleveland, OH USA; 6 Mellen Center for Multiple Sclerosis, Cleveland Clinic, Cleveland, USA

**Keywords:** Depression, Suicide, Suicidality, Multiple sclerosis, Privacy, Patient management

## Abstract

**Abstract:**

Multiple sclerosis (MS) causes physical, emotional, and cognitive changes that impact function and quality of life (QoL). Risk factors for suicidality in MS patients include a high incidence of depression, increased isolation, and reduced function/independence.

**Purpose of Review:**

To describe the epidemiology of depression and suicidality in this population, highlight warning signs for suicidal behavior, provide recommendations and resources for clinicians, and discuss ethical decisions related to patient safety vs. right to privacy.

**Recent Findings:**

Fifty percent of MS patients will experience a major depression related to brain MRI factors and disease-related psychosocial challenges. Nevertheless, depression is under-recognized/treated. The standardized mortality ratio (SMR) indicates a suicide risk in the MS population that is twice that in the general population.

**Summary:**

Given the prevalence of depression and the increased risk of suicide in the MS population, any clinician providing care for these patients must be prepared to recognize and respond to potential warning signs.

## Introduction

Multiple sclerosis (MS) is a chronic, immune-mediated disease that affects the white and gray matter in the central nervous system, causing a wide range of unpredictable and often disabling physical, cognitive, and emotional symptoms [1]. Depression, one of the most common symptoms of MS, increases the risk of suicidal ideation and death by suicide in this population [2, 3]. It is essential therefore for clinicians in every discipline to be equipped with practical recommendations for dealing with a suicidal MS patient, whether they work as part of a team in an MS comprehensive care setting or as a practitioner in a solo or group practice. In this paper, we will review the epidemiological data relating to suicide, discuss how to respond to suicidal ideation and behavior in a variety of clinical settings, highlight the ethical challenges related to confidentiality and mandatory reporting, and address ways to handle personal feelings and reactions when dealing with suicidal intent or death by suicide in one’s patients.

## A Word About Terminology

The language we use when talking about suicidality conveys a great deal about our attitudes and feelings. Table 1 provides definitions of common terms and suggests language that is both more direct and less value-laden (See Table 1)

**Table 1:** Suicide terminology

Definitions [4]	
*Suicide*: a fatal, self-injurious act associated with at least some intent to die *Suicidality*: a term that includes suicidal ideation, suicide planning, suicide attempts, and deaths by suicide	
*Suicidal behaviors* • Suicidal ideation: thoughts of engaging in behavior intended to end one’s life • Suicide plan: formulation of a specific method through which one intends to die • Suicide attempt: engagement in potentially self-injurious behavior associated with at least some attempt to die (distinguished from non-suicidal self-injury, in which there is no intent to die)	
*Value-laden terminology*	*Suggested replacement terminology*
“Commit” suicide or “successful” suicide attempt	“Death by suicide” or “suicide”
“Completed” suicide or “successful” suicide	“Died by suicide”
“Unsuccessful” or “failed” suicide attempt	“Non-fatal suicidal behavior”

## Setting the Stage for a Conversation About Suicidality in Our Patients

“There is no more emotive topic in medicine than suicide. In an age of evidence-based medicine in which outcomes are scrutinized, mulled over, fiercely debated, and disagreed upon, suicide stands apart. Here, there is unanimity – it is the outcome no one wanted, not even the deceased, we reason, for surely such an extreme behavior is the product of a disordered mind with judgment overcome by pain, depression and despair.” [5••]

“We need to address the root causes of our nation’s suicide problem….and for those at risk who still slip past all the checkpoints, we need to make sure they don’t have access to guns and lethal medications….If we ignore all this, and keep telling the story that there is a simple solution at hand, the families of suicide victims [and their healthcare providers] will be left wondering what they did wrong.” [6]

Any discussion of suicide in people with MS (PwMS) must acknowledge the importance of depression as a major causative factor in thoughts and acts of self-harm. While it is clear that factors other than depression are also implicated, the profound degree to which sadness and its companion, anhedonia, can affect the lives of PwMS, makes it an appropriate starting point for understanding why, to some, life no longer seems worth living.

Depression is common in PwMS. The incidence per 100,000 people is 979, equal to that of anxiety, bipolar affective disorder, and psychosis combined, all of which have elevated rates relative to the general population^.^ [2]. Put another way, it is estimated that up to one in two PwMS will develop a major depression over the course of their lifetime [3]. The data suggesting that depression is more common in women than men with MS are equivocal, unlike the clear female preponderance in the general population [7]. The reasons for the high rates of depression in MS are diverse. Structural MRI has revealed associations with regional brain lesion volume [8], atrophy [9], and subtle changes in normal appearing brain tissue [10]. Functional MRI has revealed abnormal prefrontal-subcortical network connectivity not only in depressed PwMS but also in euthymic MS individuals when challenged to process emotion-laden stimuli. The latter finding, not present in healthy individuals [11], hints at why some people with the disease are potentially vulnerable to depression. Brain MRI metrics can account for approximately 40% of the variance in the presence of depression [10], a percentage matched by a constellation of psychosocial factors such as poor coping strategies, uncertainty, and the loss of hope [12]. These data suggest that for certain individuals with depression, one must look beyond the reductionist imaging model to a more complex interplay of factors in order to understand the origins of their mood change.

The effects of depression on the lives of PwMS are widespread and significant. Depression is a major determinant of quality of life [13]. When severe, it can exert a negative effect on cognition by reducing cognitive capacity [14] and slowing processing speed [15]. Given the high prevalence of depression in PwMS, these observations take on a pressing clinical relevance. The morbidity and mortality associated with depression in PwMS should, however, be viewed alongside data showing that depression is treatable. Cognitive behavior therapy has been endorsed by the America Academy of Neurology as the treatment of choice for depression in PwMS [16], while benefits have also been reported with antidepressant medication [17].

Suicidal intent may be the harbinger of a suicide attempt. A number of studies have explored this in PwMS. Two of these studies utilized the Patient Health Questionnaire (PHQ-9), a nine item self-report scale that mirrors the symptoms that define major depression according to the Diagnostic and Statistical Manual of Mental Disorders (5th Ed. DSM5). In the first of these, 8.5% of 188 participants had thoughts of self-harm over a 2-week period, the figure increasing threefold when the timeframe was extended to 6 months. Factors other than depression associated with these self-injurious thoughts were age over 65 years, poor coping skills, and greater physical limitations with bladder, bowel, swallowing, and speech [18].

A second PHQ-9 study with a sample of 445 PwMS reported that almost one in three participants had experienced *transient* thoughts of suicide during a 2-week period, with depression and bowel dysfunction emerging as significant predictors. Notably, prevalence of *persistent* suicidal thinking during this period was 10.8% and was predicted by depression only [19]. A third study, which used a structured clinical interview to assess psychopathology in a sample of 140 PwMS in a tertiary care clinic, found that 28.5% of participants had had thoughts of suicide since the diagnosis of MS. The advantage of a structured interview lies in its ability to generate psychiatric diagnoses. Major depression, anxiety disorders, co-morbid depression and anxiety disorders, and alcohol use disorder were all associated with suicidal thinking, as were a family history of mental illness and living alone. Three potentially modifiable variables, namely, major depression, alcohol abuse, and living alone correctly predicted 85% of participants with thoughts of suicide. The challenges faced by patients and caregivers here were starkly revealed by an additional set of statistics. A third of suicidal individuals had received no psychological assistance while two thirds with a major depression, all suicidal, were not receiving therapy [20].

As further corroboration of suicidal ideation in people living with MS, the National MS Society’s MS Navigator® service receives approximately 140 suicide-related calls per year. Prominent themes include financial burdens, lack of emotional support, depression, worsening symptoms, difficulty with activities of daily living, and care partners coping with suicidal loved ones [21].

When it comes to death by suicide, a number of different methodologies have been employed to assess whether the risk is elevated in PwMS. The preferred method is the standardized mortality ratio (SMR), which refers to the ratio of the observed number of deaths in PwMS over a given period to the number of deaths expected in the general population during the same period. SMR factors in an age correction, an important consideration given the increased rate of death by suicide in middle age and in the very elderly [22]. SMRs can be reported together with confidence intervals and statistical significance values, although not all studies utilize these. Their usefulness is underscored by the observation that an SMR of greater than 1.0, indicative of an elevated risk of suicide, may still not be significant statistically.

A series of epidemiological studies from Scandinavian countries utilizing large population-based databases have revealed generally consistent SMR findings indicating an increase in suicide in PwMS. For example, three studies looked at data from the Danish Multiple Sclerosis Registry that spanned the 1950s through the 1990s and reported SMRs that ranged from 1.62 to 2.12 [23–25]. Swedish data confirm these conclusions during a similar time period irrespective of whether the results were based on an SMR [26] or a hazards ratio, the latter adjusting for demographic and timeline factors [27•]. These results are offset to a limited degree by a Finish study, which reported an SMR of 1.7 (95% CI 0.9–2.7), but this was not statistically significant [28] and a Norwegian study that failed to find an elevated SMR, perhaps because suicide data were not distinguished from those pertaining to accidental death [29]. These two studies apart, the conclusions from a meta-analysis confined to SMR data point to an elevated suicide rate in PwMS [30••]. On the other hand, studies utilizing a variety of methodologies apart from SMR have reached a more equivocal conclusion [5••].

A number of suggested risk factors for suicide in PwMS have either been refuted or are still awaiting definitive replication. With respect to the former, an early report of treatment with interferon β leading to an increase in depression, and by association suicide and attempted suicide [31], has not been confirmed [32]. With the occasional exception, it is now recognized that post-treatment depression is more closely linked to a past history of depression than the drug per se [33]. More concerning is a potential association between suicide and younger males relatively soon after receiving the diagnosis. While it is known that women are more likely to attempt suicide than men, men are more likely to die by suicide [27•]. More clarity is needed related to the window of greatest risk post diagnosis. One study suggests the first year [25], whereas others extend this to 5 years [26, 28, 34]. What also remains unknown is the frequency with which suicidal intent, an established risk factor for death by suicide, is followed by an attempt. Findings from a tertiary care center study revealed that while 28.5% of PwMS had thought of suicide, 6.4% had attempted it and survived [20]. The complexity of this relationship, however, is borne out by the observation that not all individuals who had attempted self-harm had thought about it beforehand. Impulsive behavior comes with its own set of predictors that, within a MS population, still require defining.

## Recognizing the Warning Signs

Seventy-seven percent of people who die by suicide have had contact with a physician in the year prior to their death and 40% have been seen within their last week [35]. Being sensitive to potential warning signs, and prepared to address them, are therefore essential.

*Letter from a patient to his neurologist*

1) I decided to have no more treatment. I am tired of being sick. 2) I am tired of living in assisted living or now independent living where dependent kids visit there [sic] parents who are older than me. I’m tired of being depressed and living in assisted living. I asked to move to ____ in a small apartment and everyone said no. So, I decided I have the solution it will make sense to me. Have a good life.

P.S. I tired of being in depressing environments. I did not write my daughters because they have my cell phone # and address but nobody ever ever sent me card or phone call ever. How depressing life is.

*Statements made during a physical therapy session*

“I don’t want to be a burden anymore….”

“I hate my life….”

“I can’t do anything, so why should I live…?”

“My life is terrible…I just want to be done with all of this.”

*Statements made to physical therapist by a care partner*

“I’m worried that she sees no other life than her old one before MS”

“He tells me he has lived a good life – he seems ok if this all ended.”

Potential red flags include:

- Positive depression screen
- Abrupt changes in health and/or behavior (e.g., stopping exercise, becoming less socially active, putting affairs in order, increasing alcohol intake)
- Intense, ongoing bereavement
- Statements of hopelessness
- Social isolation
- Substance abuse
- Worry about being a burden
- Inadequate support system
- Concerned family members

## Responding to Red Flags in the Comprehensive Care Setting

It is important to respond in a direct and proactive way to verbal or behavioral red flags.

- Assess your patient’s safety and environment
- Reinforce your therapeutic relationship by:
  - Scheduling more frequent visits, including family or friends where possible
  - Limiting refills on medications that could be harmful
  - Referring promptly for mental health services to treat depression, anxiety, and substance abuse
- Ask specific and direct questions about:
  - Passive thoughts of self-harm (e.g., “I would rather not be here.”)
  - Previous attempts to harm oneself
  - Access to firearms (availability doubles the risk of dying by suicide)
  - Access to other lethal means

## Cleveland Clinic Mellen Center for MS—Suicidality Protocol [36]

- Review PHQ-9 results, patient report, and your perceptions
- Do not leave patient unattended
- Call Social Work or Psychiatry for support
- Complete form for involuntary transport to the Emergency Dept.
- Transport patient by EMS or police
- Call ER to inform them of the transfer
- Finish/sign treatment note ASAP to facilitate psychiatry access in the ER
- Early and careful follow-up
  There is no evidence that asking about suicide increases the risk of suicide. A proactive conversation about suicidal thoughts and feelings can save a person’s life; avoiding the conversation benefits no one.

- In the event of hospitalization for suicidality, follow up with your patient promptly and plan for continuity of care [37].
  - 43% of deaths by suicide occurred within a month of discharge
  - 47% of PwMS died before the first follow-up appointment

## Responding to Red Flags in a Private Practice (Non-mental Health) Setting

Clinicians such as rehabilitation professionals, who spend large amounts of time with their MS patients but have little or no mental health training, may hear or see red flags during their patient visits.

### Patient Example

A 36-year-old male with primary progressive MS transitions to female, with mental health services in place during the gender-reassignment process. She returns to PT after surgery because of increasing disability requiring full-time wheelchair use. She is no longer able to live alone and questions the surgery and her will to live this life she “imposed” on herself.

### Care Partner Example

A care partner tells the PT after a therapy session that her 65-year-old husband with progressive MS—a retired airline pilot who is confined to a wheelchair and fully dependent on her for all daily activities—has said that he is tired of living. The wife is terrified and asks the PT what she should do.

It is important to feel prepared for these types of situations by:

- Being familiar with HIPAA and state law compliance requirements
- Including the requirement to report in your Notice of Privacy Practices
- Informing the patient when there has been a significant infringement on his or her privacy if the patient would not otherwise be aware
- Considering the use of a depression screening tool with all your patients (e.g., PHQ-9 or PhQ-2, Columbia suicide scale?)
- Planning for unexpected conversations (see Table 2)
- Identifying community and professional resources that can assist you (e.g., mental health colleagues, local law enforcement, local EMS responders) and to whom you can make referrals

**Table 2:** Recommendations for in-the-moment conversations

Do say	Avoid saying
o I am glad you feel comfortable sharing with me…. o Depression is a very common symptom of MS o I am concerned for you and care about you. May I connect you with someone who can help…? o For your safety, I need to contact	o Nothing o This is going to be OK…. o I get depressed sometimes, too…. o Do not worry about it, this feeling will pass…. o Let us take your mind off it with some good stretching/exercises

Regardless of the treatment setting, a team-based approach is the most beneficial for your patient and for you. Be familiar with the local crisis centers and helplines and build connections with mental health professionals in the community to whom you can refer patients.

## In the Aftermath of a Death by Suicide in your Practice

The stigma is powerful and real, and feelings of guilt, fear, anger, and avoidance are common. Talking with others who have been through a similar situation can be invaluable for both family members and healthcare professionals.

- It *is* helpful to review the case for any red flags, missed clues, or opportunities for handling the situation differently.
- It *is not* helpful to assume responsibility for a patient’s actions or to harbor guilt or a sense of failure.

## Ethical Challenges in Practice

Clinicians across all practice settings face ethical challenges regarding suicidal ideation or behaviors in their patients, particularly given that patient safety increases as confidentiality decreases.

*Health Insurance Portability and Accountability Act* (*HIPAA*) [38]

Exception allows for breaking of confidentiality when disclosure of personal health information

“…is necessary to prevent or lessen a serious and imminent threat to the health or safety of a person or the public; and is to a person or persons reasonably able to prevent or lessen the threat….”

- When are we permitted to break confidentiality to communicate with other caregivers or a patient’s family about suicidal thoughts or behaviors?

Clinicians are generally permitted to break confidentiality when, according to that clinician’s professional judgment, patient expressions of suicidality present a “serious and imminent threat to health or safety” [38]. This allows clinicians to communicate with other caregivers, a patient’s family, law enforcement, or other persons reasonably able to prevent or lessen the threat.” [38]

- What is the scope of our professional obligations when patients express suicidal ideation or intent?
  While no federal laws mandate reporting of suicide risk for a given capacitated adult patient, obligations to report suicidality derive from clinical protocols, professional ethics rules, and/or state laws. US state laws vary as to which licensed professionals—including health professionals, mental health professionals, educators, and law enforcement personnel—may be required to report suicidal intent or behavior.
  Each clinical discipline has its own code of ethics. It is essential that each of us is familiar with that code and knows the implications for dealing with suicidal patients. Similarly, the scope of professional obligations will differ somewhat by clinical discipline—i.e., more inclusive for those in mental health and medical fields and more circumscribed for those in rehabilitation or other fields. Therefore, being confident about where our professional obligations lie will help us feel more confident about our actions.

## When Dying with Dignity Is a Patient’s Priority

The conversation about suicide in patients with MS must acknowledge death with dignity as a rational choice for some individuals. While being vigilant regarding suicidality in our patients, we must also be open to conversations with those whose quality of life has been diminished by progressive debilitating disease to the point that they see little reason to continue living. These patients may want to discuss options for dying a dignified death on their own terms—a choice that is legal in some countries and some states in the US.

Clinicians should familiarize themselves with the legal requirements in their area of practice and, at the same time, carefully assess their own feelings and attitudes about physician-assisted dying.

It is important to refer these patients for a psychological assessment to ensure that undiagnosed or untreated symptoms of a mental health disorder, such as depression, are not playing a role in the decision-making process. At the same time, making sure that everything possible has been done to manage symptoms and optimize quality of life will minimize the chances of a person ending her or his life prematurely.

For areas in which physician-assisted dying is not legal or for patients who do not meet legal requirements, clinicians may still be able to assist the patient in their focus on quality of life through palliative medicine referral or cessation of treatment.

## Recommended Resources

- National Suicide Prevention Lifeline: 1-800-273-TALK (8255)
- Zero Suicide Toolkit: http://zerosuicide.sprc.org/toolkit
- Mental Health America: www.mentalhealthamerica.net
- Means Matter website, from the Harvard T.H. Chan School of Public Health http://www.hsph.harvard.edu/means-matter/
- SAFE-T Pocket Card for Clinicians – Five-step evaluation and triage for suicide assessment
- Suicide Prevention and the Clinical Workforce: Guidelines for Training from the Clinical Workforce Preparedness Task Force of the National Action Alliance for Suicide Prevention
- Clinical Practice Guideline for Assessment and Management of Patients at Risk for Suicide, from the Department of Veterans Affairs, Department of Defense, June 2013.
- http://www.sprc.org/: Suicide Prevention Resource Center

## Conclusions and Key Practice Points

- People with MS can have a variety of risk factors for suicide.
- Awareness of possible red flags can help you identify at-risk patients.
- Ask specific and direct questions about passive thoughts of self-harm, previous attempts at self-harm, access to firearms and other lethal means.
- There is no evidence that talking about suicidal thoughts with patients increases their risk of suicide.
- Do not go it alone. Work with your in-house team and/or create a team in your community that includes other health professionals, law enforcement, and relevant agencies and resources.
- Care partners and healthcare providers need support and compassion in the aftermath of a death by suicide.
